# Homeopathic medical practice: Long-term results of a cohort study with 3981 patients

**DOI:** 10.1186/1471-2458-5-115

**Published:** 2005-11-03

**Authors:** Claudia M Witt, Rainer Lüdtke, Roland Baur, Stefan N Willich

**Affiliations:** 1 Institute for Social Medicine, Epidemiology and Health Economics, Charité University Medical Center, D-10098 Berlin, Germany; 2 Karl und Veronica Carstens Foundation, Am Deimelsberg 36, D-45276 Essen, Germany

## Abstract

**Background:**

On the range of diagnoses, course of treatment, and long-term outcome in patients who chose to receive homeopathic medical treatment very little is known. We investigated homeopathic practice in an industrialized country under everyday conditions.

**Methods:**

In a prospective, multicentre cohort study with 103 primary care practices with additional specialisation in homeopathy in Germany and Switzerland, data from all patients (age >1 year) consulting the physician for the first time were observed. The main outcome measures were: Patient and physician assessments (numeric rating scales from 0 to 10) and quality of life at baseline, and after 3, 12, and 24 months.

**Results:**

A total of 3,981 patients were studied including 2,851 adults (29% men, mean age 42.5 ± 13.1 years; 71% women, 39.9 ± 12.4 years) and 1,130 children (52% boys, 6.5 ± 3.9 years; 48% girls, 7.0 ± 4.3 years). Ninety-seven percent of all diagnoses were chronic with an average duration of 8.8 ± 8 years. The most frequent diagnoses were allergic rhinitis in men, headache in women, and atopic dermatitis in children. Disease severity decreased significantly (p < 0.001) between baseline and 24 months (adults from 6.2 ± 1.7 to 3.0 ± 2.2; children from 6.1 ± 1.8 to 2.2 ± 1.9). Physicians' assessments yielded similar results. For adults and young children, major improvements were observed for quality of life, whereas no changes were seen in adolescents. Younger age and more severe disease at baseline were factors predictive of better therapeutic success.

**Conclusion:**

Disease severity and quality of life demonstrated marked and sustained improvements following homeopathic treatment period. Our findings indicate that homeopathic medical therapy may play a beneficial role in the long-term care of patients with chronic diseases.

## Background

Homeopathy is one of the most frequently used and controversial systems of complementary and alternative medicine. It is based on the 'principle of similars', whereby highly diluted preparations of substances that cause symptoms in healthy individuals are used to stimulate healing in patients who have similar symptoms when ill [1]. When a single homeopathic remedy is selected based on a patient's total symptom picture, it is called 'classical' homeopathy [2]. According to a survey in the US [3], the proportion of patients obtaining homeopathic care has quadrupled in the last seven years. A survey in Britain [4] estimated that annual expenditures reached £34.04 million (out-of-pocket £30.74 million, NHS £3.3 million). For Germany, the country in which classical homeopathy originated, a recent survey demonstrated that approximately 10% of men and 20% of women in the general population used homeopathic medicines during the previous year [5]. General trends show a rise in the number of individuals utilising naturopathic and homeopathic therapeutic methods [6].

The General Medical Council in Germany grants an official certification in homeopathy to physicians upon successful completion of a three-year-long training programme. Approximately 4,500 physicians in Germany hold this additional certification [6]. However, with the exception of some randomised, controlled trials including patients with selected diagnoses [2,7] there is no data on the health care offered by classical homeopathic medical practices. Therefore, it is impossible to assess the state of homeopathic health care and its effectiveness. We designed this project with the goal of systematically collecting data in the area of homeopathic health care for the first time in Germany. The aim of the present study was to determine the spectrum of diagnoses and treatments, as well as the course of disease over time among patients who chose to receive homeopathic treatment.

## Methods

Patients were included consecutively in this prospective, multi-centre observational study upon their first consultation with a participating physician and were followed up for a total of 24 months. Evaluations were made using standardised questionnaires. In order to provide as representative a picture of homeopathic health care as possible, patients were included in the study regardless of their diagnosis. Patients were eligible for the study if they were consulting the participating physician for the first time and were at least 1 year of age. In order to participate in the study, physicians were required to have passed certified training in classical homeopathy and at least three years of experience in its practice. A total of 187 physicians belonging to four different working groups were contacted either by post or telephone and informed about the study. Of these, 103 physicians chose to participate. Each participating physician was trained in study procedures and was subject to at least one monitoring visit during the study period. All study participants provided written, informed consent, and the study protocol was approved by the appropriate ethics review boards.

### Outcome measures

For patients, we developed different questionnaires for three different age groups: 1–6 years of age, 7–16 years of age, adults (>16 years of age). All questionnaires were designed to document sociodemographic data, as well as information on prior medical history, patient symptoms and complaints, quality of life, and the use of any treatment other than homeopathy. At baseline, patients recorded the complaints that led them to consider homeopathic treatment. Independently of their physicians, patients rated the severity of their complaints on a numeric rating scale (0 = no complaints, 10 = maximum severity) [8]. All complaints listed by patients in their baseline questionnaire were transferred to their follow-up questionnaires by the study office personnel. This ensured that each baseline complaint was assessed at each subsequent follow-up. For children between 1 and 6 years of age, the KITA questionnaire [9] was used to assess general health-related quality of life. It was completed by the children's parents. Patients between 7 and 16 years of age completed the KINDL questionnaire [10,11]. In additional, parents were asked to provide the required medical information. For the adults, general health-related quality of life was assessed using the MOS SF-36 questionnaire [12]. The results of the SF-36 are presented in normalised scores, the results being scaled in such a way that the normal German population has a mean score of 0 and a standard deviation of 1.

The first questionnaire was distributed to the patients by the study physician and completed prior to the start of therapy (baseline). Patients sent their completed questionnaires to the study office in sealed envelopes. Follow-up questionnaires were sent to all patients by the study office at 3, 12, and 24 months.

For physicians, we developed a standardised questionnaire that allowed for continuous documentation during the treatment/follow-up period (24 months), as well as standardised points of assessment at 0, 3, 12 and 24 months. At each of these time points, the severity of a maximum of 4 diagnoses and maximum of 8 symptoms was rated by participating physicians using a numerical rating scale [8]. This information was then forwarded to the study office. The type of homeopathic treatment, the use of any conventional therapy, as well as any referrals to other physicians were recorded on a continuous basis.

### Statistics

Data was double entered manually into an ACCESS^© ^database and subsequently compared using the SAS^© ^system followed by plausibility data checks if necessary. The diagnoses, documented by study physicians, were encoded in ICD-9 format and recorded by two specially trained study staff members using DIACOS.^© ^Statistical analysis was performed using SAS/STAT^© ^software (Version 8.2).

Data for adults (>16 years) and for children/adolescents were analysed separately. In order to calculate the average severity of the physicians' diagnoses, we took the four diagnoses named first for each patient during the baseline examination. For each of the follow-up assessment points (i.e. at 3, 12, and 24 months) we ascertained the respective severity ratings made by study physicians.

All results reported here are based on the intention-to-treat approach, i.e. each included patient entered the final analyses. If patients dropped out or withdrew from the study we replaced the respective missing values: baseline complaints that had been cured were given a severity rating of 0 in all following examinations. For patients who died during the study, we inserted the maximum severity rating of 10. Other missing values were multiply imputed following the suggestions of Rubin [13]. Instead of filling in a *single *value as a substitute for a missing value, multiple imputation is a strategy by which each missing value is replaced simultaneously by a *set *of plausible values that represents the uncertainty about the right value to impute. Thus, the missing values are filled in several times generating several distinct data tables, each with a complete set of data without any missing value. These complete data tables are analyzed separately using appropriate statistical models. Afterwards, the results from all statistical analyses are pooled to generate treatment effects and p-values. In our study we used the MCMC (Marcov chain Monte Carlo) replacement method and created 5 multiple imputed data tables.

For each imputed data set, treatment effects were estimated on the basis of generalized linear regression models. Generalized linear regression models are flexible and powerful tools to describe data from cohort studies [14]. They are generalizations of the well known and often applied multiple regression models which often appear to be too simple to describe longitudinal data adequately. A generalized linear model is best described by two components. First, the mean course of the outcome, and second, the correlation structure for measurements taken at the same individual at different times. In our study we divided the 2-year follow-up period into two parts. During the first part (months 0–3) we assumed that mean outcome increases (or decreases) linearly. For the second part (months 3–21) we assumed that the mean outcome increases (or decreases) according to a quadratic term. Moreover, we assumed that the correlation between two measurements can be described by a simple exponential function. This essentially means, that the correlation only depends from the time span between the two measurements, and it decreases the bigger this time span is. This approach is completely analogous to the recommendations given by Diggle, Liang, and Zeeger in their standard text book on the analysis of longitudinal data [14].

Subgroup analyses are based on essentially the same statistical approach adding the respective factors as a fixed covariate into the models. For subgroup analyses adults' and children's data were pooled.

Usually, patients for clinical studies are not selected randomly from a target population but according to some selection criteria that sample patients according to extreme measurements (high blood pressure, severe pain, low quality of life, ...). This inevitably leads to regression-to-the-mean, a statistical phenomenon that makes natural variation look like real changes [15]. Separating regression-to-the-mean effects from true treatment effects can be difficult but is at least feasible when the mean and the standard deviation of the target population are known. In this situation it is possible to calculate the expected outcome for each patient when regression-to-the-mean occurs [16]. In our study we made a rather conservative assumption on the target population (chronically ill patients seeking homeopathic care): to have the same quality of life as the general German population (i.e. a mean SF-36 score of 0 and a standard deviation of 1). From this we calculated the expected regression-to-the mean effect and compared it to the actually observed change of the SF-36 scores.

## Results

A total of 103 physicians participated in the study (51 male, 45 ± 7 years of age; 52 female, 45 ± 7 years of age). Twenty-six percent of the participating physicians were specialists (10% internists, 9% paediatricians, 7% other) and 74% were general practitioners. The average duration of overall medical practice was 17.4 ± 8.4 years with 9.0 ± 4.4 years of practice in homeopathy (range 3–20 years). Forty percent of the physicians were certified to work in the public health care system, and 60% were in private practice.

**Figure 1:**
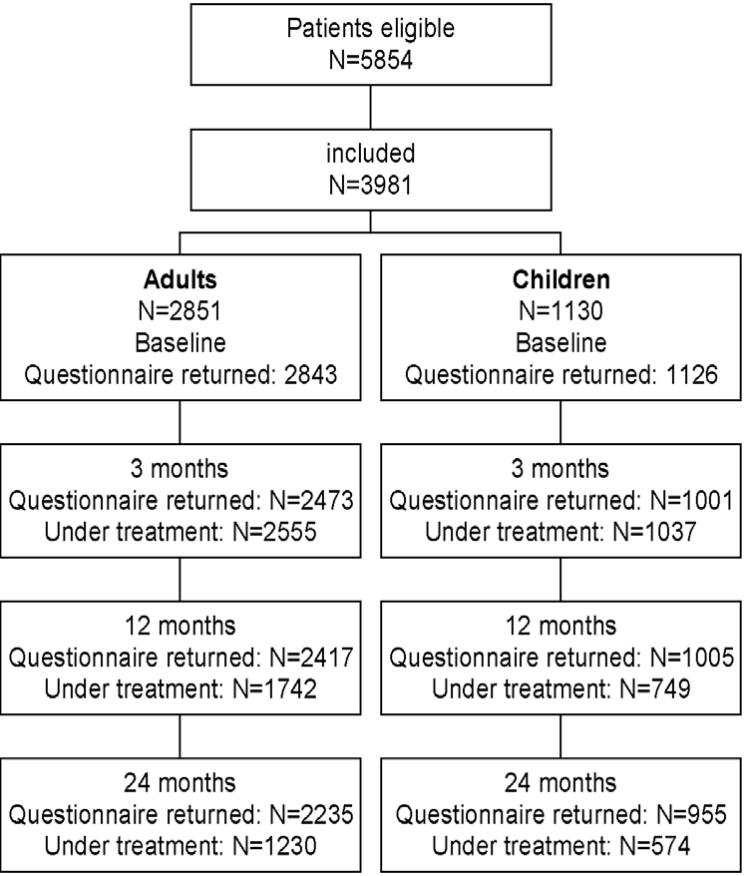
Patient selection.

Patients were recruited for the study between September 1997 and December 1999. Of the patients who met the inclusion criteria, 3981 (68%) chose to participate and were included in the study (for patient selection see Figure 1). Of these, 2851 were adults (71% women) and 1130 were children (48% girls). The baseline characteristics are listed in Table 1.

**Table 1:** Baseline characteristics of study population

	Adults	Children
Gender (% female)	70.8	48.3
Age (years, mean ± std)	40.7 ± 12.7	6.7 ± 4.1
Marital status (% living in partnership)	84.0	/
Education (% attending school >10 years)	85.0	/
Belief in homeopathy (%)	65.7	68.6^†^
Duration of disease (years, mean ± std)	10.3 ± 9.8	4.3 ± 3.7
Intake of conventional drugs (%)	50.2	31.7

† Parents' perspective

**Figure 2:**
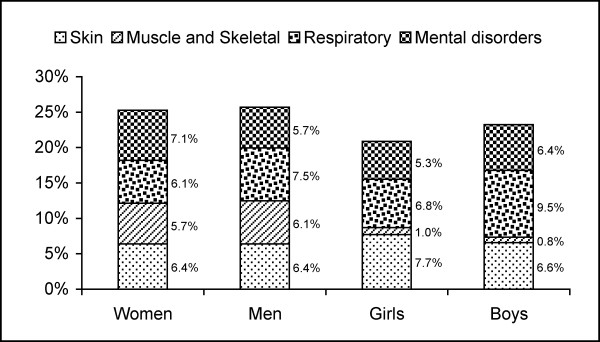
Most common medical complaints as reported by the homeopathy physicians (in % of documented complaints).

On average, the homeopathic physicians made 2.6 ± 1.2 diagnoses per patient (2.8 ± 1.1 in adults, 2.3 ± 1.1 in children). Ninety-seven percent of all diagnoses were classified by these physicians as chronic with a median duration of 4.3 ± 2.7 years in children and 10.3 ± 9.8 years in adults. Almost all patients had received conventional treatment (95%) or had already contacted another physician (95%) prior to the start of this study. The most common diagnosis in women was migraine (9.7%), in men allergic rhinitis (10.3%), and in children of both genders atopic dermatitis (20%), for details see [17]. For the most common disease groups see Figure 2.

All patients underwent an initial homeopathic anamnesis, lasting an average of 2.0 ± 0.7 hours. Following enrolment in the study, patients had to wait an average of 57 ± 84 days before undergoing the initial anamnesis. During the 24-month observation period, patients consulted their physicians an average of 7.8 ± 8.4 times. During the study period, half of the patients (50.3%, adults: 50.8%, children 48.9%) noted additional visits to non-study physicians (gynaecologists and dentists excluded). The intake of conventional medication decreased from 45.0% at baseline (adults: 50.2%, children 31.7%) to 26.8% after 24 months (adults: 31.8%, children 14.2%).

According to patient assessments, disease severity decreased significantly between baseline and 12 months, as well as between 12 months and 24 months (see Table 2). According to physician assessments, 25.7% (adults: 21.9%, children: 37.6%) of the diagnoses were no longer present at 24 months, whereas patients judged 23.0% (adults 19.7%, children 32.8%) of the medical complaints to have resolved by this point. Thirteen percent of the patients documented that they had no complaints whatsoever at 24 months.

**Table 2:** Course of outcome parameters and estimated mean changes of outcome parameters

					**Estimated changes compared to baseline†**		
	**Baseline**	**3 months**	**12 months**	**24 month**	**Δ 3 months**	**Δ 12 months**	**Δ 24 months**
	mean ± SD	mean ± SD	mean ± SD	mean ± SD	mean (95%CI)	mean (95%CI)	mean (95%CI)
**Adults**							
Patients assessments (NRS) ‡	6.2 ± 1.7	3.8 ± 2.2*	3.3 ± 2.1*	2.9 ± 2.2*	-2.4 (-2.5 to -2.3)	-2.8 (-2.9 to -2.7)	-3.1 (-3.2 to -3.0)
Physicians assessments (NRS) ‡	6.0 ± 1.6	3.9 ± 2.1*	2.8 ± 2.1*	2.1 ± 2.0*	-2.1 (-2.2 to -2.0)	-3.1 (-3.2 to -3.0)	-3.7 (-3.8 to -3.6)
SF-36 QoL physical scale	46.5 ± 10.1	49.1 ± 9.5*	50.1 ± 9.6*	50.7 ± 9.5*	2.6 (2.3 to 2.9)	3.5 (3.0 to 3.9)	4.1 (3.5 to 4.6)
SF-36 QoL mental scale	39.3 ± 11.8	44.6 ± 10.8*	45.5 ± 10.8*	46.4 ± 10.6*	5.6 (5.2 to 6.0)	6.2 (5.7 to 6.7)	6.9 (6.3 to 7.6)
**Children**							
Patients assessments (NRS) ‡	6.1 ± 1.8	3.2 ± 2.3*	2.5 ± 2.1*	2.2 ± 2.0*	-3.1 (-3.3 to -2.9)	-3.5 (-3.7 to -3.4)	-3.9 (-4.0 to -3.7)
Physicians assessments (NRS) ‡	5.9 ± 1.7	3.2 ± 2.2*	2.0 ± 1.5*	1.5 ± 1.8*	-2.7 (-2.8 to -2.6)	-3.8 (-4.0 to -3.7)	-4.4 (-4.6 to -4.3)
KINDL QoL	69.3 ± 13.3	72.1 ± 12.6	68.0 ± 9.2	67.3 ± 9.9*	2.7 (1.7 to 3.7)	-0.4 (-1.5 to 0.8)	-2.2 (-3.6 to -0.8)
KITA QoL mental/physical dimension	67.6 ± 16.9	75.4 ± 14.6*	77.0 ± 14.1*	77.5 ± 14.3*	8.3 (6.6 to 10.0)	9.3 (7.7 to 10.8)	10.0 (8.3 to 11.6)
KITA QoL aspects of daily living	58.6 ± 18.3	66.9 ± 15.9*	69.1 ± 16.7*	70.6 ± 16.0*	8.5 (7.2 to 9.8)	10.4 (8.8 to 12.0)	11.6 (9.7 to 13.5)

† estimations are based on generalised linear models, see text; ‡ = lower values indicate better status and negative Δ indicates improvement

QoL = quality of life; NRS = numeric rating scale, * p < 0.001 versus baseline

In adults, large improvements in quality of life were observed on both component scales (mental and physical) during the first three months of treatment, and continued to improve during the course of the study (see Table 2). Even with the pessimistic assumption that the test-retest correlation of the SF-36 is only 0.7 and that the study population is no more ill than a random sample of the general population, one could expect an improvement of only 3.8 (1.2) score points on the mental (physical) component scale, attributable to regression-to-the-mean [16], markedly lower than the 5.6 (2.6) score points observed in our study (Table 2). Statistically, the baseline quality of life of non-completers was not significantly lower than in other patients (p-values: MCS: p = 0.37; PCS: p = 0.48, Wilcoxon-tests).

Quality of life in young children (age 1–6 years) also improved markedly during the observation period (Table 2), having already risen during the first three months of study therapy as measured on both scales of the KITA questionnaire (mental-physical dimension and aspects of daily living, each p < 0.001, see Table 2). These improvements continued over the course of treatment (p < 0.001, see Table 2). In school children and adolescents, however, an improvement in quality of life was only visible during the first three months of study therapy (p < 0.001, see Table 2).

The diagnosis had no relevant influence on the changes in patient complaints or quality of life as measured in this investigation.

In patient and physician assessments, younger patients showed greater improvements than did older patients and more severe disease at baseline was followed by greater improvements compared to less severe disease (see Table 3). Gender, duration of disease and belief in homeopathy had only a minor influence on improvements.

**Table 3:** Subgroup analyses for patients and physicians assessments (mean changes of outcome parameters after 24 months compared to baseline, negative Δ indicates improvement)

	Patients assessments (NRS)			Physicians assessments (NRS)		
	Mean†	95%-CI	p value*	Mean†	95%-CI	p value*
**Total **(n = 3981)	-3.3	-3.4 to -3.2		-3.9	-4.0 to -3.8	0,060
**Gender**						
Female (n = 2560)	-3.4	-3.5 to -3.2		-3.9	-4.0 to -3.8	
Male (n = 1412)	-3.3	-3.4 to -3.1	0.387	-3.9	-4.0 to -3.8	0,060
**Age groups (years)**						
<10 (n = 839)	-4.0	-4.2 to -3.8		-4.4	-4.6 to -4.2	
10–19 (n = 355)	-3.5	-3.7 to -3.2	<0.001	-4.3	-4.5 to -4.0	0.149
20–39 (n = 1456)	-3.4	-3.6 to -3.3	<0.001	-3.7	-3.8 to -3.6	<0.001
40–59 (n = 1041)	-2.8	-3.8 to -2.0	<0.001	-3.6	-3.8 to -3.5	<0.001
≥ 60 (n = 281)	-2.6	-2.9 to -2.2	<0.001	-3.5	-3.8 to -3.2	<0.001
**Baseline severity of disease**						
NRS < 6.0 (n = 1660)	-2.1	-2.3 to -2.0		-3.1	-3.2 to -3.0	
NRS ≥ 6.0 (n = 2310)	-4.1	-4.2 to -4.0	<0.001	-4.6	-4.7 to -4.5	<0.001
**Duration of disease in adults (years)**						
< 10 (n = 1878)	-3.2	-3.4 to -3.1		-3.7	-3.8 to -3.6	
≥ 10 (n = 927)	-2.9	-3.1 to -2.7	<0.001	-3.6	-3.7 to -3.4	0.043
**Intake of conventional drugs at baseline**						
Yes (n = 1788)	-3.3	-3.5 to -3.2		-3.8	-3.9 to -3.7	
No (n = 2188)	-3.3	-3.5 to -3.2	0.157	-3.9	-4.0 to -3.9	0.029
**Belief in homeopathy**						
Strong (n = 2656)	-3.4	-3.5 to -3.1		-3.9	-4.0 to -3.8	
Weak (n = 1316)	-3.1	-3.3 to -3.0	<0.001	-3.8	-3.9 to -3.7	0.563

† estimations are based on generalised linear models, NRS = numeric rating scale; * per item each subgroup compared to the first listed subgroup

## Discussion

Patient and physician assessments of disease severity and quality of life consistently demonstrated substantial improvements following homeopathic treatment, which were maintained through 24 months' follow up. Improvements were more pronounced in younger patients and in those with greater disease severity compared to older patients and those with less severe disease at baseline.

To our knowledge, the present study is the first to evaluate systematically the range of diagnoses and therapies in classical homeopathic medical practices in Germany and Switzerland. In addition, the study provided information on the course of illness in patients receiving homeopathic treatment, as assessed by patients and physicians.

The methodological strengths of our study include consecutive enrolment of a large sample size, the participation of approximately 2% of all physicians certified to practice homeopathy in Germany and 28% of all members of the Hahnemann Association (an organisation for physicians practicing only 'classical' homeopathy) and the use of standardised outcome instruments also used in studies on conventional therapy.

One limitation of our study is that the observed effects cannot be categorized with respect to specificity, i.e. we cannot draw conclusions as to the beneficial mechanisms. Furthermore patients were allowed to use conventional therapies during the study period in addition to homeopathic treatment. Thus, the observed improvement cannot be attributed to homeopathic treatment alone. The aim of the investigation, however, was not to test the effectiveness of homeopathic treatment alone, but rather provide systematic and detailed information about the current status of homeopathic medical care in routine practice and its effectiveness. These data may also be helpful in the planning of further research projects on homeopathy.

The effects observed by patient and the physician assessment, as well as those seen with regard to quality of life, deserve additional comments. The average severity of the chronic diseases was reduced by approximately 50% after only 3 months of homeopathic treatment, and remained around this level during the follow-up period. Physician assessments tended to be more positive than patient assessments.

The improvements we observed in our patients cannot be attributed solely to regression-to-the-mean, because the improvements were greater than could be expected even under conservative model assumptions. This is supported by the fact that patients did not visit the study physicians when they were feeling the worst, but rather after a long waiting period.

A strength of this study is that patients with all diagnoses were included. Therefore, no disease-specific measurement instruments could be used. To assess the severity of different medical complaints, there is no other generally accepted measuring instrument available. Instead numerical rating scales [8] were applied, which would allow for the determination of illness severity in a diagnosis-independent manner.

Compared to the other quality two of life questionnaires used in our study, the KINDL questionnaire for the age group 7 to 16 years was not sensitive to change, as has been shown in other studies [18,19]. Other explanations might be that children adapt easier to perceived quality of life and that the dimensions of Quality of life used for adults are not transferable to children. However, there is no other generally accepted measuring instrument available in German-speaking countries.

In the range of baseline diagnoses, chronic illnesses clearly predominated (>95% of diagnoses). Among these, headache and atopic disease (allergic rhinitis, asthma and atopic dermatitis) were the most common diagnoses. As the clinical histories of our patients showed, most of our patients decided to consult a homeopathic physician only after having received conventional treatment. This, together with the extensive initial case taking and the reputation of homeopathy as a "medicine designed to treat the individual as a whole"' causes a selection for chronic illnesses.

We were unable to confirm the common notion that homeopathy is frequently used for trivial complaints or diseases. The duration of disease in study patients was very long and their symptoms were, on average, of moderate severity.

In this study we were not able to evaluate different types of homeopathic strategies. For quality assurance purposes, we avoided selecting a random sample of homeopathic physicians for the study, choosing instead to recruit physicians schooled and certified in 'classical' homeopathy. The results of our study are, therefore, representative only for the classical type of homeopathy that was practised by participating physicians. Compared to conventional medical practices, headache and atopic disease (allergic rhinitis, asthma and atopic dermatitis) were the most common diagnoses in homeopathic practices (as opposed to hypertension, hyperlipidemia and low back pain in 70,000 patients treated conventionally) [9]. An American study [20] found asthma, depression, otitis media, and allergic rhinitis to be the most common diagnoses treated in homeopathic practices, compared to hypertension, upper respiratory tract infection, otitis media and diabetes mellitus, which were treated most commonly in conventional practices.

A health insurance company project that included about 900 patients treated with homeopathy in routine care [21] showed an improvement in quality of life and in physician assessment. In Güthlin's study [21], however, only physicians certified to work in the public health care system were able participate. Homeopaths working in private practices (i.e. the great majority in Germany) were excluded. The advantage of the present study is that doctors in private practice were also included, thus providing a more detailed and broader basis for describing the current status of homeopathic health care. Another controlled study in cooperation with a German health insurance company [22], indicated similar overall effectiveness of homeopathically versus conventionally treated patients for selected diagnoses and in some groups, superiority of homeopathic treatment.

## Conclusion

We evaluated for the first time the range of diagnoses and therapies at medical practices offering classical homeopathic treatment in Germany and Switzerland. The findings of our study demonstrate that patients who seek homeopathic treatment are primarily those suffering from long-standing, chronic disease. Both according to physician and patient assessments, the severity of complaints decreased markedly over the 24-month observation period. Younger patients and those with more severe disease appear to benefit most from homeopathic treatment. Among adults and children, we observed an increase in quality of life. Our findings indicate that homeopathic medical therapy may play a beneficial role in the long-term care of patients with chronic diseases.

## Competing interests

The author(s) declare that they have no competing interests.

## Authors' contributions

CW participated in the design of the study, coordination and statistical analysis. RL participated in its design and performed the statistical analysis. RB participated in the design of the study and data acquisition. SNW conceived of the study, and participated in its design and statistical analysis and had the overall scientific responsibility. All authors helped to draft the manuscript, read and approved the final manuscript.

## Pre-publication history

The pre-publication history for this paper can be accessed here:


